# Placental transcriptome co-expression analysis reveals conserved regulatory programs across gestation

**DOI:** 10.1186/s12864-016-3384-9

**Published:** 2017-01-03

**Authors:** Sam Buckberry, Tina Bianco-Miotto, Stephen J. Bent, Vicki Clifton, Cheryl Shoubridge, Kartik Shankar, Claire T. Roberts

**Affiliations:** 1 The Robinson Research Institute, The University of Adelaide, School of Paediatrics and Reproductive Health, Adelaide, 5005 Australia; 2 University of Western Australia, Harry Perkins Institute of Medical Research, Perth, 6009 Australia; 3 University of Western Australia, Australian Research Council Centre of Excellence in Plant Energy Biology, Perth, 6009 Australia; 4 The University of Adelaide, School of agriculture, food and wine, Adelaide, 5005 Australia; 5 University of Arkansas for Medical Sciences, The Department of Pediatrics, Little Rock, 72202 USA

**Keywords:** RNA-seq, Placenta, Preeclampsia, Co-expression, Gene expression, Microarray

## Abstract

**Background:**

Mammalian development *in utero* is absolutely dependent on proper placental development, which is ultimately regulated by the placental genome. The regulation of the placental genome can be directly studied by exploring the underlying organisation of the placental transcriptome through a systematic analysis of gene-wise co-expression relationships.

**Results:**

In this study, we performed a comprehensive analysis of human placental co-expression using RNA sequencing and intergrated multiple transcriptome datasets spanning human gestation. We identified modules of co-expressed genes that are preserved across human gestation, and also identifed modules conserved in the mouse indicating conserved molecular networks involved in placental development and gene expression patterns more specific to late gestation. Analysis of co-expressed gene flanking sequences indicated that conserved co-expression modules in the placenta are regulated by a core set of transcription factors, including ZNF423 and EBF1. Additionally, we identified a gene co-expression module enriched for genes implicated in the pregnancy pathology preeclampsia. By using an independnet transcriptome dataset, we show that these co-expressed genes are differentially expressed in preeclampsia.

**Conclusions:**

This study represents a comprehensive characterisation of placental co-expression and provides insight into potential transcriptional regulators that govern conserved molecular programs fundamental to placental development.

**Electronic supplementary material:**

The online version of this article (doi:10.1186/s12864-016-3384-9) contains supplementary material, which is available to authorized users.

## Background

The placenta is the first human tissue to start developing once the embryo implants into to the mother’s uterus shortly after conception. At implantation, placental trophoblast cells begin to invade into the lining of the uterus, where they colonise and transform the mother’s spiral arteries and the extra-embryonic tissue placental tissue establishes its own network of blood vessels. Together these processes facilitate the exchange of all nutrients, gases and waste throughout pregnancy. Normal placental function is dependent on appropriate growth and development of its structural components, which are underpinned by the fine-tuned regulation of gene expression. Consequently, alterations to placental gene regulation are thought to be a major contributor to pregnancy pathologies. Several studies aimed at elucidating the molecular basis of placental development have utilised high-throughput gene expression technologies, such as RNA sequencing (RNA-Seq) and microarrays, and show that the placenta undergoes global shifts in gene expression across human gestation [1–4]. They also show that placentas from pre-eclamptic pregnancies feature a distinct expression signature [5–9], and that some of these expression differences arise approximately six months before the condition manifests [10]. Recently, two placental transcriptome studies employing RNA-Seq have described the breadth of gene expression in the human placenta and show that the placenta exhibits unique patterns of exon splicing and greater than four-fold enrichment for >800 genes compared to other human tissues [11, 12].

A common feature in previous studies on placental gene regulation is that expression data are typically summarised at the gene level for between-group comparisons, widely known as *differential expression*. With differential expression, the greatest significance is attributed to individual genes where the differences between groups reach an appropriate significance threshold. Although differential expression analyses have unquestionable utility, the inherent natural organisation of the transcriptome remains largely unexplored. Conversely, co-expression analyses that consider the gene-wise relationships in gene expression data have cast new light on previously unappreciated patterns of transcriptional organisation with regards to processes and functions such as lipid metabolism [13], cancer [14], human brain development and neuropathology [15–17], and embryonic development [18]. Gene co-expression analyses identify groups of genes where expression levels are highly correlated across samples. By leveraging the inter-individual expression variability between biological samples, a co-expression analysis can enable the identification of higher-order relationships among genes. Further *post hoc* characterisation of these relationships can then provide insight into the biological processes arising from the underlying transcriptional program. Therefore, to gain a new perspective on placental genome regulation across human gestation and between human and mouse, we performed a comprehensive analysis of placental gene co-expression.

## Results

### RNA sequencing

To explore patterns of gene co-expression in the healthy human term placenta, we performed single-strand 100-base paired-end total RNA-Seq for 16 samples, obtaining a total of 1.32 billion paired reads with and average of 83 million reads per library. The mapping rate was 94.6 ±16.6% with an average of 26.2 ±8.8 million uniquely mapped pairs per library overlapping annotated genes (Additional file 1: Figure 1a). By summarising the RNA-Seq reads by counting the number of overlaps with hg19 genes (see “Methods”), we detected 15,861 genes (including both coding and non-coding RNAs) above the threshold of >1 read count per million, which we show is an accurate threshold of detection based on quantification of spiked synthetic RNAs (Additional file 1: Figure 1b and c). The normalised gene expression values were also highly correlated (Additional file 1: Figure 2), with a Pearson’s correlation coefficient for each pair being 0.97 ±0.01.

### Constructing a weighted human placental co-expression gene network

**Fig. 1:**
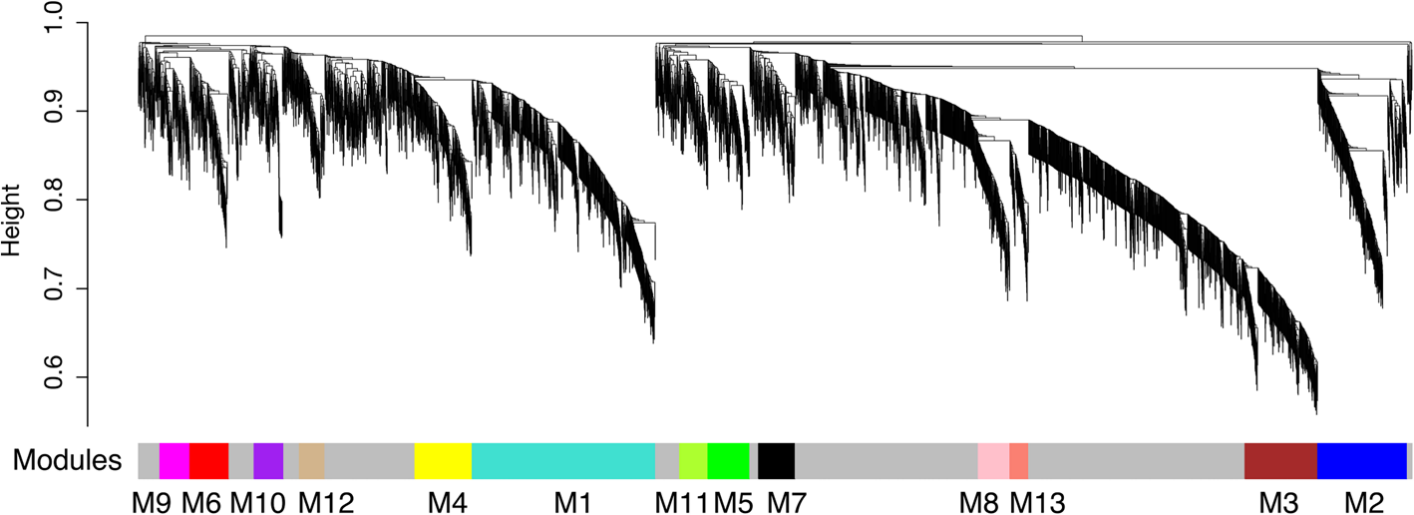
Weighted gene co-expression network analysis of the human placenta reveals distinct clusters of co-expressed genes. Weighted gene co-expression network analysis of the human placenta reveals distinct clusters of co-expressed genes. Average linkage hierarchical clustering dendrogram of genes based on gene expression topological overlap. Modules of co-expressed genes were assigned colours and identifiers M1–M13, which are represented in the horizontal bar below the dendrogram

To integrate gene-level expression profiles into a higher-order systems level framework, normalised gene expression values were used to perform a weighted gene co-expression network analysis (WGCNA) [19]. To construct the gene-wise network, we first calculated Pearson’s correlation matrix, then raised this matrix to a power to weight strong correlations at the expense of weaker ones, thus resulting in a weighted network (see Methods). To identify groups of genes with highly correlated patterns of expression, these data were then transformed into a topological overlap matrix of ‘connection-strengths’ [19]. This was then used as input for unsupervised hierarchical clustering, where we employed a dynamic tree-cutting algorithm [20] to group tree branches into 13 distinct clusters of highly connected genes, which we refer to as *modules* (Fig. 1).

Each module was then summarised by calculating the module *eigengene* for each sample, which is the first principal component of gene expression values for the module. Therefore, the eigengene represents a weighted average of gene expression. For each gene, we then define its membership in each module as the absolute correlation between the gene’s expression and the module’s eigengene, and represent this correlation as *kME* [19]. Genes are assigned to modules if they have an absolute *kME*>0.7. Note that by quantifying membership through correlation, module membership for each gene is no longer binary and allows genes to be members of more than one module (Additional file 1: Figure 3), thus connecting modules in a network.

**Fig. 2:**
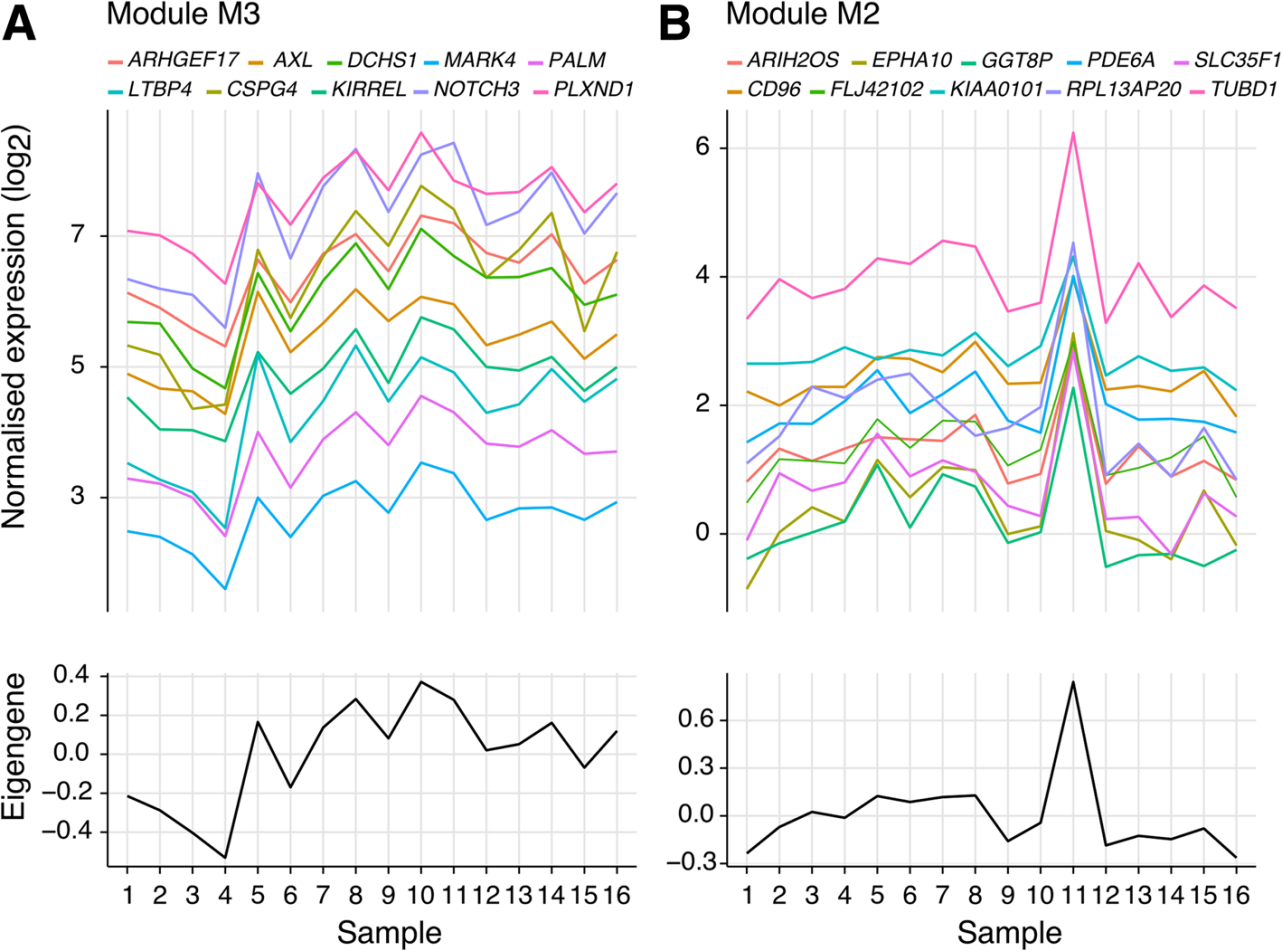
Gene–eigengene correlations identify module hub genes that are consistently co-expressed in the human placenta. Gene–eigengene correlations identify module hub genes that are consistently co-expressed in the human placenta. The upper line plots show the top ten genes with the highest module membership (*kME*) for modules M3 (**a**) and M2 (**b**). Each continuous line represents a gene, with different genes showing a similar variability of expression across samples on the x-axis

The proportion of gene expression variation explained by each eigengene ranged between 39.1% (M10) and 79.6% (M3) (Table 1). This demonstrates that even for large modules such as M3 (844 genes), a significant proportion of variance can be captured by a single representative value. For each gene module, the top hub genes (*kME*>0.9) are reported in Table 1, and genes with a *kME*>0.7 for each module are listed in Additional file 2. The plots in Fig. 2 demonstrate the high correlation of the top ten most connected genes for modules M2 and M3, and how gene variance is accurately reflected by the module eigengene.

**Table 1:** Co-expression module characteristics

Module	No. of genes	Variance explained by eigengene	Top ten hub genes (*kME*>0.9)
M1	740	44.6%	*ZNF845, ZNF808, GPR160, GIN1, ATP5J, ZNF567, ANAPC10, C8orf59, MRPS36, RBM7*
M2	262	48.9%	*EPHA10, ARIH2OS, TUBD1, FLJ42102, KIAA0101, RPL13AP20, CD96, PDE6A, GGT8P, SLC35F1*
M3	844	79.6%	*NOTCH3, PLXND1, PALM, CSPG4, ARHGEF17, DCHS1, MARK4, KIRREL, LTBP4, AXL*
M4	566	51.5%	*HMMR, CASC5, DEPDC1, CDK1, KIF15, CCNA2, AIM1, TTK, ESCO2, EXO1*
M5	116	45.5%	*ATP2A1, C11orf35, P2RY2, CCDC33, ASIC3, KIFC2, IL17REL, CLIC3, MTVR2, RBBP8NL*
M6	88	51.4%	*HN1, ASAP3, SLC12A8, ASPHD2, B3GNT7, IL17RE, PRG2, NOG, IL2RB, PIPOX*
M7	112	41.5%	*SNORD114-29, CDH11, FAM198B, SNORD114-7, SNORD114-10, FKBP7, SNORD114-14, C4orf32, SNORD114-26, SNORD113-2*
M8	390	68.1%	*SBF1, ULK1, STRA6, DOT1L, BCAR1, TMEM184A, B3GNT8, SLC25A22, C19orf71, INTS1*
M9	79	44.5%	*SELL, S100A12, LRRK2, CYTIP, MNDA, ACSL1, FPR2, TGFA, LOC100505806, TMEM71*
M10	110	39.1%	*MTHFS, TTTY15, RPS4Y1, TXLNG2P, TTTY10, KDM5D, UTY, EIF1AY, ZFY, PRKY*
M11	112	43.1%	*PGAP3, GPR137, PRR5, ARTN, C10orf10, C7orf43, ALDH4A1, EFS, RELL2, ADIRF*
M12	81	51.7%	*PVRL4, ARHGEF4, NDRG1, INHBA, SYDE1, INHA, MIR210HG, C8orf58, SIGLEC6, PDZD7*
M13	414	71.0%	*FAM195B, FBXL15, BRAT1, AKAP2, SCAND1, EME2, CCDC85B, C19orf60, PGLS, TSR3*

As our dataset featured equal number of samples from male and female fetuses, we expected that at least one co-expression module would be correlated with fetal sex status and would serve as a positive control. To test this, we performed a chromosomal enrichment test which identified module M10 to be significantly enriched for Y chromosome genes (Fisher exact test, Bonferroni *p*=2.9×10^−12^, *OR*=29.4, Additional file 1: Figure 4). Accordingly, M10 eigengene expression was also significantly higher for male samples (t-test, *p* =3.5×10^−5^, *CI*=0.27−0.57, Additional file 1: Figure 5).

As placental gene expression has previously been shown to be influenced by method of delivery and the onset of labor [21], we tested for an association of delivery method (operative vaginal, unassisted vaginal and cesarean section) and found no significant associations for any co-expression module (ANOVA tests with Bonferroni correction, all *p*>0.05). We further tested for eigengene correlations with birthweight and gestational age at delivery and found that M3 eigengene expression was moderately correlated with birthweight (Pearson’s *r*=0.53, Student asymptotic *p*=0.035, Additional file 1: Figure 6), however this correlation failed to remain significant after Bonferroni correction.

### Co-expression modules are reproducible

**Fig. 3:**
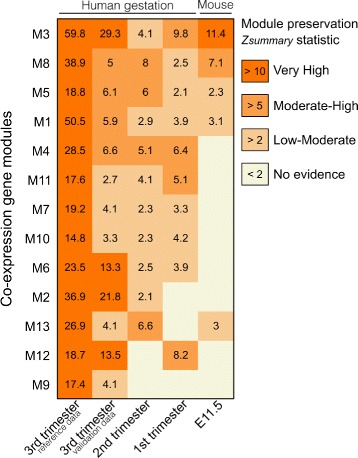
Preservation heat map of co-expression gene modules in independent datasets shows level of module preservation in the human placenta across human gestation and in mid gestation mouse placenta (E11.5). Colours represent four classes of co-expression preservation as represented by Z-score summary of preservation statistics. *Z*_*summary*_>10 indicates high level of evidence for module preservation, *Z*_*summary*_ 5-10 indicates moderate-high preservation, *Z*_*summary*_ 2-5 indicates low-moderate preservation, and *Z*_*summary*_<2 indicates no evidence for preservation. Numbers within cells are the *Z*_*summary*_ statistic. Third trimester reference (far *left* column) represents results from running permutation tests using the data collected in this study. Third trimester validation data (n=20) is from ref [11]. Second trimester gene expression data (n=27,GSE5999) is from ref [2]. First trimester expression data (n=16, GSE28551) is from ref [1]. Mouse expression data at E11.5 (n=23, SRA062227) is from ref [24]

To evaluate the reproducibility of these gene modules in the third trimester placenta, we utilized RNA-Seq data from a previously published study on the human placental transcriptome [11] and tested whether the density and connectivity patterns of gene modules we defined in our reference dataset were preserved. To quantify reproducibility, we applied a preservation permutation test [22] to summarise evidence that the network topology is preserved in independent test sets and report the *Z*_*summary*_ statistic to summarise module preservation. In this independent third trimester dataset, 4/13 modules show highly significant preservation scores *Z*_*summary*_, and 8/13 were at least preserved *Z*_*summary*_>5 despite a lower depth of sequencing [11] (Fig. 3). A gene ontology analysis showed that conserved co-expression modules such as M3 and M8 are enriched for distinct biological processes fundamental to placental development such as cell adhesion and vascular system development (Additional file 3).

### Key co-expression modules are preserved across human gestation and conserved in the mouse

Given that the human placenta undergoes significant growth and remodeling throughout the nine months of gestation [23], we reasoned that if particular co-expression modules were involved in core placental functions, then these modules would be reproducible using gene expression data from earlier gestational time points. To test this hypothesis, we obtained microarray gene expression data from placental tissue collected during the first (GSE28551) [1] and second trimesters (GSE5999) [2]. Although these datasets contain expression data for substantially fewer genes after filtering and annotation (57.6% and 63.9% of detectable genes in the RNA-Seq dataset, respectively), the module preservation statistics indicate that a majority of modules are nevertheless preserved across gestation at a low to moderate level of significance (Fig. 3). In particular, M4 shows moderate preservation *Z*_*summary*_>5 across all gestational time points, indicating a conserved pattern of gene regulation throughout human gestation. In contrast, the M2 module is highly preserved in the third trimester datasets *Z*_*summary*_>10 with little to no evidence of preservation during the first or second trimesters, suggesting M2 genes constitute a molecular program more specific to third trimester placental functions.

**Fig. 4:**
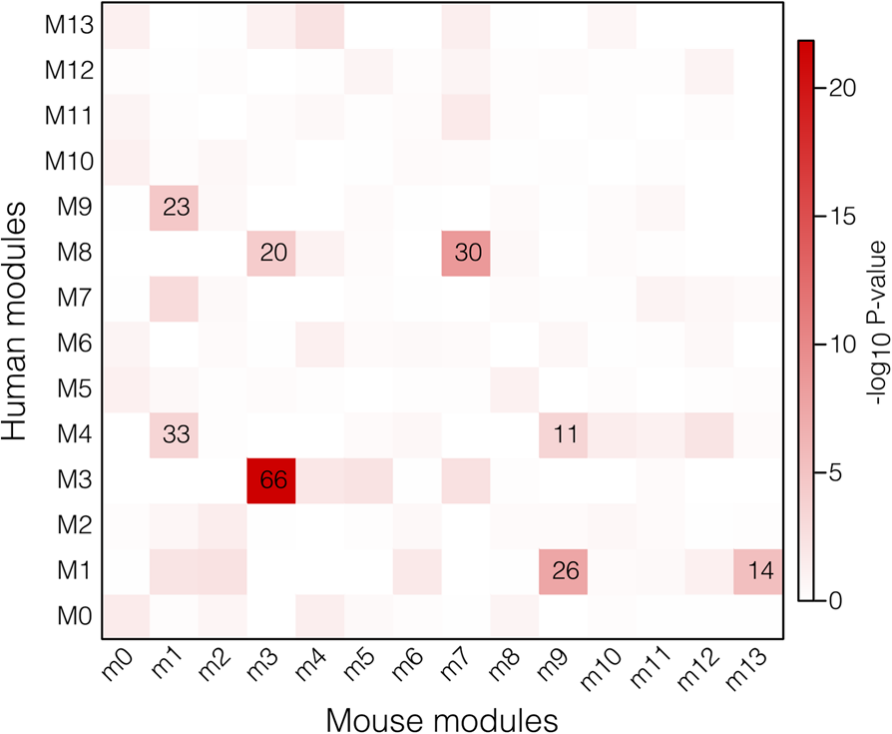
Overlap between weighted gene co-expression network modules for human and mouse placenta. Heat map colours represent Fisher exact test − log10*p*-values. Numbers within cells represent the number of overlapping genes with Bonferroni *p*<0.05 and shows five human co-expression modules (M1, M3, M4, M8 and M9) have a significant corresponding module in the mouse. The M0/m0 modules represent the groups of genes that were not assigned to any modules and are therefore not included in the networks. Mouse data is from SRA062227, ref [24]

As the mouse is the most widely utilised model for studying placental development, we next asked whether the co-expression gene modules were conserved between human and mouse. To achieve this, we obtained raw RNA-Seq data (SRA062227) for 23 mid-gestation (E11.5) mouse placenta samples [24] and showed that 5/13 had some degree of evidence for module preservation *Z*_*summary*_>2, with M3 showing a highly significant preservation score *Z*_*summary*_>10 (Fig. 3). To further validate the conservation of co-expression between human and mouse, we assembled an independent and unsupervised *de novo* mouse co-expression network using the same methods as our human dataset. By counting the overlapping genes for each module and performing Fisher exact tests, we show that five human modules have at least one mouse counterpart (Bonferonni corrected *p*<0.05, Fig. 4). As predicted from the human–mouse *Z*_*summary*_ statistics, M3 showed the highest degree of overlap with a mouse module (Bonferroni *p*=2.78×10^−20^) and a highly significant *kME* correlation (Pearson’s *r*=0.4, *p*=2.6×10^−102^).

### Preserved modules feature a core set of transcription factor motifs

**Fig. 5:**
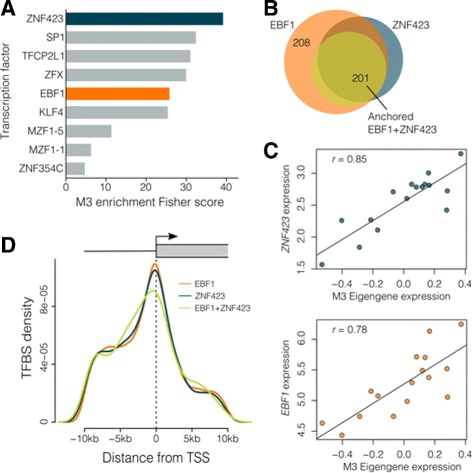
EBF1 and ZNF423 are potential upstream regulators of M3 gene expression. EBF1 and ZNF423 are potential upstream regulators of M3 gene expression. **a** Enrichment test for TF-binding motifs in the 10kb up- and down-stream of transcription start sites identify two TFs, ZNF423 (*blue*) and EBF1 (*orange*), that are members of the M3 module. **b** EBF1 and ZNF423 are predicted to target many of the same M3 genes. Circles in the Venn diagram represent the number of genes targeted by TFs and their overlap – EBF1 (*orange*), ZNF423 (*blue*), and when both have motifs directly adjacent to each other (anchored analysis, *yellow*). **c** ZNF423 and EBF1 expression is highly correlated with M3 eigengene expression. Points represent individual samples. **d** TF-binding motif density is greatest immediately upstream of M3 transcription start sites. Coloured lines represent the density TF motifs for EBF1 (*orange*), ZNF423 (*blue*) and the combination of both (*green*)

As several co-expression modules appeared to be highly conserved, we tested the 10kb up and downstream of genes in each module for enrichment of transcription factor binding motifs. This identified 52 transcription factors as potential regulators of co-expression (Additional file 4), several of which were detectable in the placenta at the RNA level and predicted to target multiple conserved co-expression modules. As M3 genes appeared to constitute the most highly conserved transcriptional network in this study (Fig. 3), we then further analyzed the transcription factors that were detectable in the placenta at the RNA level and predicted to target M3 genes. This identified *ZNF423* and *EBF1* which were both also members of the M3 module (*kME*=0.85 and *kME*=0.78, respectively), and highly correlated with the M3 eigengene (Fig. 5 c). ZNF423 has previously been reported to interact with EBF1 [25–28]. Here we show a majority of M3 genes with ZNF423-binding motifs also feature EBF1 motifs (Fig. 5 b), and the density of these motifs is greatest immediately upstream of M3 transcription start sites (Fig. 5 d). These multiple lines of evidence suggest ZNF423 and EBF1 are potential regulators of M3 gene transcription. When we performed the same enrichment tests for all other modules, ZNF423 and EBF1 were predicted to target a high proportion of genes within other co-expression modules (Additional file 4). Further inquiry revealed that the most highly preserved modules across human gestation, and between human and mouse (M1, M3-5, M8), feature a core set of TF-binding motifs (Additional file 1: Figure 7), suggesting these co-expressed genes share common regulatory elements and have a high degree of flanking sequence similarity.

### Modules of co-expressed genes are implicated in pregnancy complications

**Fig. 6:**
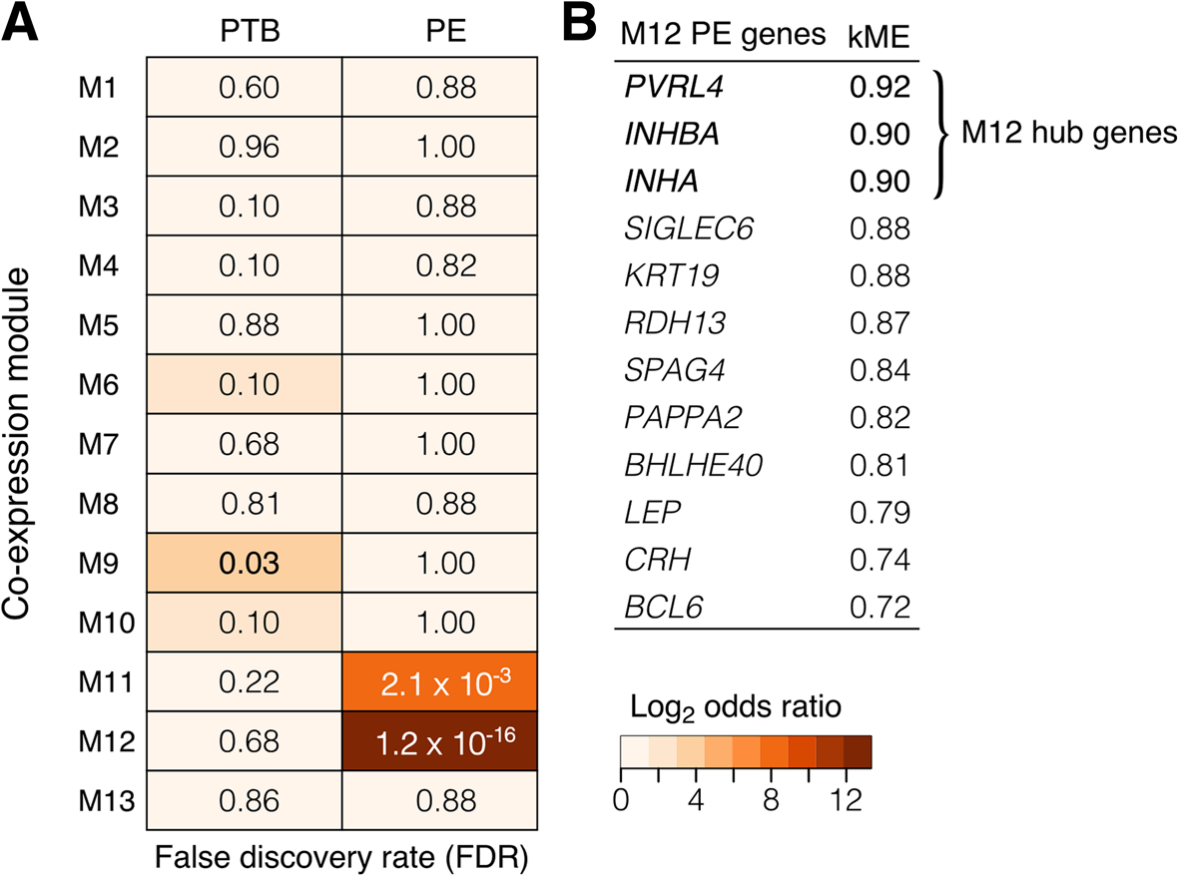
M12 is enriched for genes that show a meta-signature for preeclampsia. M12 is enriched for genes that show a meta-signature for preeclampsia. **a** Heat map table shows the statistical enrichment (FDR) of module genes in preterm birth (PTB) and preeclampsia (PE), and cell colours represent log2 odds ratio. **b** M12 genes implicated in PE and their module membership (*kME*). M12 intramodular hubs are in **bold**. PTB associated genes were obtained from PTB gene database [32] and genes with a PE signature obtained from ref [5]

The origins of several pregnancy pathologies, such as preterm birth (PTB) and preeclampsia (PE) are largely attributed to abnormal placental development [29–31]. If co-expression modules constitute gene networks involved in placental development, we reasoned that if a particular module underpinned key placental processes, it may be enriched for genes implicated in pregnancy complications. To address this question, we obtained a curated gene list from the PTB gene database [32], and a set of meta-analysis-validated differentially expressed genes in PE [5], and tested our co-expression gene modules for enrichment of genes implicated in these pathologies (Fig. 6). M9 was statistically enriched for genes associated with PTB (*OR*=3.4, *FDR*=0.03), but more strikingly, modules M11 and M12 showed significant enrichment for PE-related genes (M11 *OR*=16.6, *FDR*= 2.1×10^−3^; M12 *OR*=101.3, *FDR*= 1.2×10^−16^). Notably, three M12 intramodular hub genes (*PVRL4*, *INHBA* and *INHA*) have consistently been shown to be up-regulated in PE [5]. This provided the first line of evidence that M12 gene co-expression genes may be altered in PE.

**Fig. 7:**
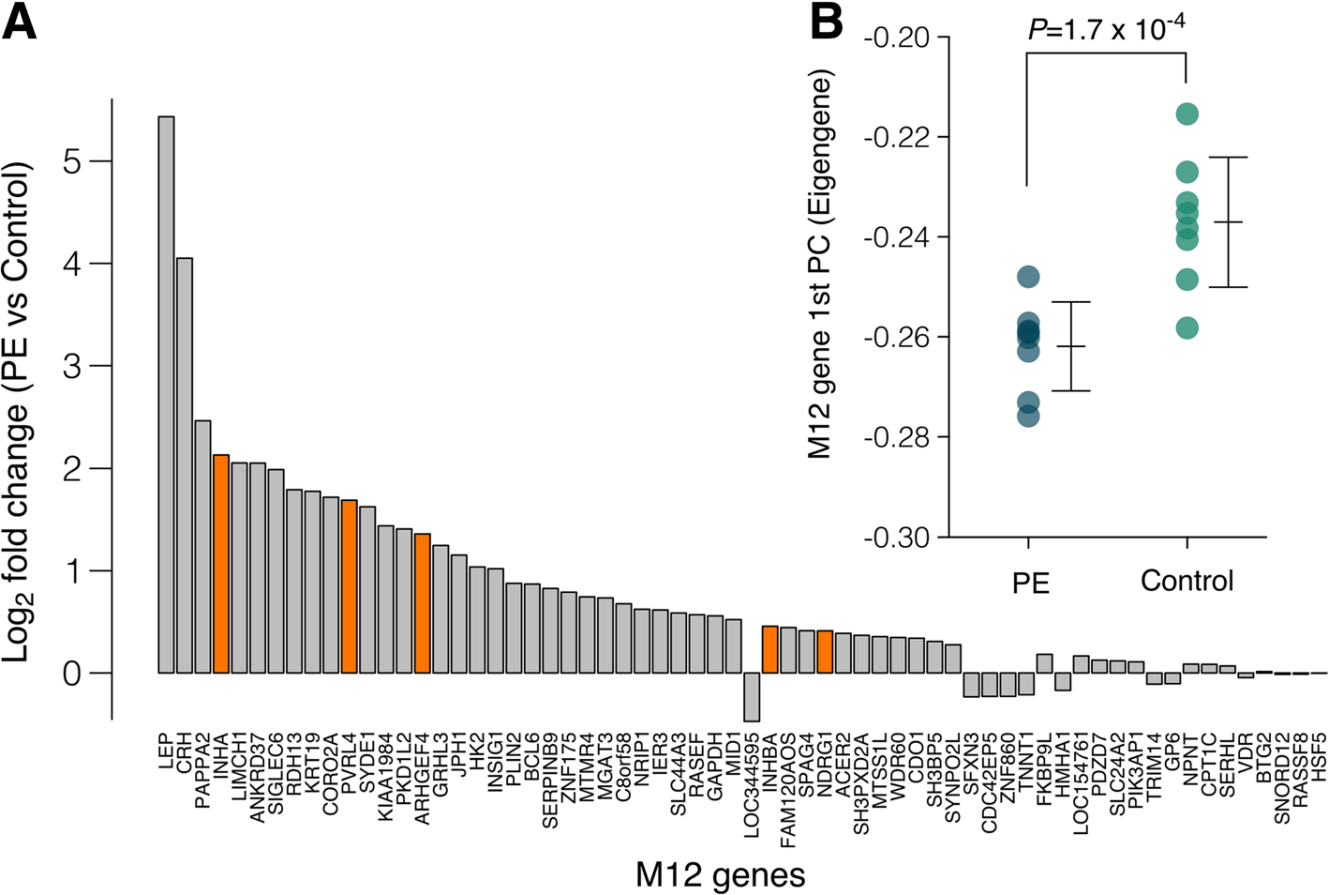
M12 genes are significantly up-regulated in preeclampsia placentas. M12 genes are significantly up-regulated in preeclampsia placentas. **a** Bar plot showing the log2 fold-change between preeclampsia and control placentas. Orange bars represent M12 hub genes. **b** The M12 eigengene (first principal component) is significantly different between preeclampsia and control placentas. Validation gene expression data obtained from GSE44711, ref [33]

To further validate the finding that M12 was enriched for genes differentially expressed in PE, we obtained additional independent microarray expression data from a recent study on early-onset PE (*n*=16) [33] and tested for differences in M11 and M12 gene expression. First, a rotation gene set test [34] showed that (61*%*) of M12 genes are significantly up-regulated in the PE placenta (*p*=0.021), with M11 showing no significant enrichment (*p*=0.938). When testing for differential expression of all genes in preeclampsia versus controls independently, 261 genes were significant (absolute fold change >2, *FDR*<0.05) with M12 showing the highest proportion of differentially expressed genes (Additional file 1: Figure 8). This independent analysis thus provides a second line of evidence for the involvement of M12 genes in preeclampsia (Fig. 7 a). Following this, we calculated the first principal component for M12 genes in this dataset to obtain an eigengene measure, and showed that M12 eigengene expression is significantly different (*t*-test, *p*=1.7×10^−4^) between PE and control (Fig. 7b). This demonstrated the robust nature of the eigengene for testing for differences in gene regulation between control and PE pregnancies. Together, these results implicate M12 co-expressed genes in PE and suggest that the mechanisms regulating M12 co-expression may be altered in PE.

## Discussion and conclusions

By conducting this comprehensive co-expression network analysis of the human placental transcriptome, we reveal previously unappreciated aspects of transcriptional organisation at the fetal-maternal interface. This analysis entailed the integration of multiple gene expression datasets and curated databases, which enabled the testing of specific hypotheses regarding placental genome regulation.

Our results demonstrate that a large proportion of the placental transcriptome is organised into distinct modules of co-expressed genes, some of which are preserved across gestation, and conserved between human and mouse. The reproducibility of these networks, constructed from independent datasets and different platforms (RNA-Seq and microarrays) suggest a fundamental modular organisation of the placental transcriptome. Moreover, our cross-species analysis demonstrates that certain aspects of human placental transcriptional organisation are highly preserved in the mouse, indicating the evolutionary conservation of molecular processes which drive mammalian placental development.

When comparing the *de novo* human and mouse networks, five genes were identified as M3/m3 intramodular hub genes (*kME*>0.9) in both species (*ARHGEF17*, *DOCK6*, MAP3K9 *OSBPL7*, and *PRR12*), demonstrating a high degree of inter-species module reproducibility. These hub genes are centrally located within the M3 module and may be critical components of the network. Of particular interest, *DOCK6* mutations in humans are associated with extreme placental angiopathy and a severely abnormal placental phenotype [35], while *DOCK6* expression is reported to be down-regulated in placentas from growth-restricted fetuses [36]. Similarly, *OSBPL7*, an oxysterol-binding protein, is also reported to be differentially expressed in placentas from preeclamptic pregnancies [37]. For genes that do not have any previously reported placental phenotype association, these could be potential novel candidates for involvement in placental development. Given the size of the M3 co-expression module, it is reasonable to expect that these genes would be involved in multiple cellular processes. The results of the gene ontology analysis do indicate that M3 genes are involved in processes such as cell adhesion, cardiovascular system development, growth-factor binding and extracellular matrix structre. Together, there results suggests that the M3 co-expression network may be involved in multiple levels of placental development and regulation.

Investigation of the TFs that potentially regulate co-expression revealed that the most preserved modules are predicted to be regulated by a core set of transcription factors, including the M3 genes *EBF1* and *ZNF423*, which potentially target a high proportion of genes in the most highly preserved modules. Although the effects of ZNF423 and EBF1 on placental gene regulation remain largely unexplored, ZNF423 appears to be a multi-functional transcription factor associated with B cell regulation, retinoic acid signalling, notch signalling, DNA damage response pathways, adipogenesis and cancer [25]. Furthermore, homozygous mutation in the homologous gene in mice (*Zfp423*) results in smaller ataxic pups who die shortly after birth [38]. This indicates a critical role for ZNF423 in development. EBF1 can act as both a transcriptional activator and repressor and has known roles in tumour suppression [39]. When EBF1 binds DNA directly as a dimer, it can activate transcription via p300-CBP co-activation [39]. In other contexts, the same DNA binding dimer in conjunction with ZNF423 can recruit the nucleosome remodelling and deacetylase (NuRD) complex, triggering EBF1-mediated transcriptional repression [39]. The observation that EBF1 and ZNF423 are co-expressed in the placenta and members of the M3 module, and their widespread targeting potential across modules of co-expressed genes indicates that these TFs are candidate key regulators of transcription in the placenta.

The identification of M12 being enriched for genes implicated in PE demonstrates the utility of a co-expression analysis for identifying genes that may respond to the pathology, or may indeed underlie its aetiology. This guilt-by-association approach, clustered genes implicated in PE (M12) in a completely unsupervised manner, suggesting expression differences in these genes are driven by a set of common factors. The observation that several M12 hub genes are up-regulated in PE, and show highly correlated patterns of expression, implies that expression of other genes within this module is likely driven by the same underlying factors, together indicating that these genes are implicated in placental development. Moreover, the M12 network is preserved in the first trimester (Fig. 3), the period where the pathogenesis of PE is considered to have its origins [40]. Furthermore, these patterns of co-expression do not appear to be conserved in the mouse. Although human and mouse placental development have many similarities, it is also important to note that mice do not develop preeclampsia. Together, these findings indicate further investigation of the involvement of M12 genes and their upstream regulators in human placental development may be a valuable way of generating new hypotheses regarding the placental origins of PE.

Concordant with our results, several M12 hub genes such as *NDRG1, INHA, INHBA* were central to both protein-protein interaction networks [41] and co-expression networks [42] implicated in PE in previous studies. Of particular interest, the intramodular M12 hub gene *PVRL4*, which is up-regulated in PE [5], is expressed more highly in the placenta compared to other human tissues [43]. *PVRL4* is a potent mediator of epithelial cell colony formation [44] and is also highly expressed in ovarian cancer tissue [45]. Furthermore, cleaved PVRL4 is elevated in the serum of patients with ovarian cancer and is correlated with *PVRL4* expression [45], suggesting that maternal serum PVRL4 may hold potential as a biomarker of PE. Together, these results suggest a potential role for M12 genes in the pathogenesis of PE.

One limitation of our study is the number of samples we have used to construct our co-expression networks, and the expression levels of some hub genes are relatively low. However, we are confident that our expression measurements are reasonably accurate at these levels as we emperically determined a threshold of detection using spike-in RNAs (Additional file 1: Figure 1). Furthermore, we have bolstered our analysis by incorporating multiple independent datasets to validate our results assess the preservation of co-expression networks. Secondly, as different placental biopsies can feature differing contributions of maternal versus fetal cells between different gestational ages and sampling methodologies, there are inherent limitations in comparing data between studies. This may be one underlying factor in driving the differences we observe between our dataset and the third trimeser validation dataset. We also recognise that the second trimester gene expression data (GSE5999) were from basal plate tissue collected from pre-term birth deliveries so they may not be directly comparable to the villous tissue data collected from uncomplicated pregnancies. Additionally, the mouse placental tissue we have re-analyzed (SRA062227) was collected at approximately mid gestation (E11.5) therefore the comparison with the late gestation human tissue should be interpreted with some caution. However, given the rarity of some of samples used in our analysis, we are of the opinion that the comparisons made still have value.

Several new questions arise from this comprehensive co-expression network analysis. Firstly, are patterns of co-expression altered in placental pathologies? Our analysis of independent expression datasets from PE placentas provide compelling preliminary evidence that M12 genes are up-regulated in PE, which warrants further investigation into the regulators of M12 genes. Secondly, what genetic and environmental factors influence co-expression? A comprehensive assessment of genotypes and environmental factors such as maternal diet has the potential to reveal drivers of placental expression variation. Thirdly, does silencing of hub genes shift module co-expression and influence placental cell phenotype and behavior? Functional studies aimed toward elucidating the biological function of co-expression modules may yield new insights into how placental development is regulated.

In summary, we show that a weighted gene co-expression network analysis can provide novel insights into the functional organisation of the placental transcriptome. To the best of our knowledge, the networks described herein have not been described previously, and emphasise that these findings could not be revealed through conventional gene-level summaries or differential expression experiments. In typical differential expression analyses, emphasis is placed on genes where the expression differences reach an appropriate level of significance. Although such experiments have contributed significantly to our understanding of placental genome regulation, the significance of each gene is typically determined in isolation, subsequently failing to connect genes in a manner that reflects the functional organisation of the transcriptome. By connecting genes in a manner that reflects underlying genome regulatory programs, we have exposed previously unappreciated aspects of the placental transcriptional landscape and provide a framework for multiple avenues of *post hoc* inquiry.

## Methods

### Ethics and consent

Ethics approval was granted by the Central Northern Adelaide Health Service Ethics of Human Research Committee (Approval #2005082) and the University of Adelaide Human Research Ethics Committee (H-137-2006). Written, informed consent was obtained from all patients.

### Sample collection

Third trimester placenta samples were collected from primiparous women with singleleton pregnancies classified as being uncomplicated by using the criteria described in reference [46]. Placenta samples were collected and dissected within one hour post-delivery at the Lyell McEwin Health Service, South Australia in accordance with our ethical approval (see ethics statement). Placental villous tissue was obtained by first taking a full-thickness sections and then removing the membranes and basal plate tissue before dissecting villous tissue from the middle of the section. No tissue or sample pooling was performed at any step. Samples of villous tissue were then incubated in RNAlater solution (Invitrogen) at 4 degrees celsius for 24 hours before being stored at -80 degrees celsius. Full sample details are listed in Additional file 1: Table 1.

### RNA sequencing

RNA was extracted from 16 placental samples using TRIzol following the manufacturer’s protocol. All samples were spiked with 96 External RNA Controls Consortium (ERCC) ExFold RNA transcripts. Ribosomal RNAs were depleted from samples using Ribo-Zero Gold and sequencing libraries were prepared using Illumina^®;^TruSeq^®;^Stranded Total RNA Sample Preparation kits. Sequencing was performed on the Illumina Hi-Seq 2500 using a 100bp paired-end protocol at the Australian Cancer Genomics Facility in Adelaide.

Sequence adapters were trimmed using AdapterRemoval with options –trimns, –minlength 20. Trimmed RNA-Seq reads were aligned to known UCSC hg19 genes and the hg19 genome using Bowtie 2 v2.1.0 and TopHat v2.0.9 with options –library-type=fr-firststrand –mate-inner-dist -20 –mate-std-dev 180. UCSC hg19 reference genome and transcriptome was obtained through Illumina iGenomes (support.illumina.com/sequencing/sequencing_software/igenome.html).

### Sequence data processing

Aligned RNA-Seq reads were summarised using the summarizeOverlaps algorithm with the UCSC known genes hg19 GTF file using the the options overlapMode=“Union”, ignoreStrand=FALSE, singleEnd=FALSE, fragments=TRUE [47] to generate a table of unique read counts per gene for each sample (this summarized data is available through NCBI GEO, GSE77085). Only genes >1FPKM were retained (15,861 genes) and count data were transformed and quantile-normalised using the Voom method [48] to produce a numeric matrix of normalised expression values on the log2 scale. All samples were processed in the same way, with all sequencing libraries created in the same batch and sequenced together. However, we nevertheless checked systematic differences between samples (Additional file 1: Figures 9 and 10) and found no evidence of batch effects or systematic shifts in gene expression.

### Network construction

To construct the network of co-expressed genes, we selected the most variable upper third of genes in the placental RNA-Seq dataset using the Weighted Gene Co-expression Network Analysis methods implemented in the WGCNA R package [19]. Briefly, gene expression values were used to construct a signed co-expression network by computing a Pearson’s correlation matrix, which is then used to compute an adjacency matrix by raising the correlation matrix to a power. We chose a power of eight, which was determined by plotting scale-free fit and mean connectivity as a function of power (Additional file 1: Figure 11) using the scale-free topology criteria outlined in [49]. By raising the absolute value of the correlation to a power, the construction of co-expression networks emphasises high correlations at the expense of low correlations [19]. The interconnectedness (topological overlap) of each gene pair was calculated using the adjacency matrix, which was then used as input for average linkage hierarchical clustering.

Gene modules were then defined as branches of the resulting clustering tree, with the branches cut into defined modules using the dynamic tree-cut algorithm [20]. Gene modules were then summarised by calculating module eigengenes, which are defined as the first principal components of the module expression profiles. As module eigengenes capture the maximum amount of variation of gene expression within a module, the eigengene is considered a representative value (or weighted average) of module gene expression [19]. For each module, the gene membership value (*kME*) is defined as the correlation between the standardised gene expression values for each gene and the module eigengene for each sample [19]. We assigned genes to modules if they had a high module membership defined as *kME*>0.7, and genes with a value below this threshold were assigned to the M0 (grey) module. Note that using this method allows genes to be members of more than one module.

### Module preservation

To evaluate the preservation of human third trimester placenta gene modules in independent placenta gene expression datasets, we used the WGCNA modulePreservation function to generate module preservation statistics [19]. These methods test whether the density and connectivity patterns of gene modules defined in our reference dataset are preserved in independent datasets. We used the *Z*_summary_ statistic to summarise the evidence for significant module preservation compared to a random sample of all network genes reiterated over 100 permutations per dataset. We adopted the thresholds suggested by Langfelder *et al* [13], who indicate *Z*_summary_<2 implies no evidence for module preservation, 2<*Z*_summary_<10 implies weak to moderate preservation, and *Z*_summary_>10 implies strong evidence for module preservation.

### RNA-seq validation dataset

We used the raw RNA-Seq reads from 20 human third trimester placenta samples as previously described in a separate analysis of the human placental transcriptome [11]. In this current study, RNA-Seq reads were aligned to the human reference genome and UCSC known genes (hg19) using Tophat 2 with the options –library-type=fr-unstranded –segment-length=18. For the mouse expression data, we obtained RNA-Seq fastq files for 23 samples from the NCBI short read archive (SRA062227). Reads were aligned to mm10 genome and UCSC known genes using Tophat2 with the options –library-type=fr-unstranded –read-mismatches 3 –read-edit-dist 3. Alignment bam files were summarised to obtain the number of unique read counts per gene using the summarizeOverlaps function in the genomicAlignments R package [47] with the options ignore.strand=TRUE, paired=FALSE, mode=“union” followed by log2 counts per million transformation and quantile normalisation. To enable the comparison of human and mouse datasets, mouse gene identifiers were converted to orthologous human gene identifiers using Ensembl Biomart and the biomaRt R package. Only mouse genes with one-to-one orthologues in the human dataset were included and mouse genes with no corresponding human gene were removed from the analysis.

### Microarray validation datasets

For second trimester placenta, Affymetrix CEL files for 27 samples (GSE5999) were pre-processed, background subtracted and normalised using the robust multi-average (RMA) algorithm [50]. Pre-processed and normalised data from 16 first trimester placenta samples (GSE28551) and third trimester preeclampsia samples (GSE44711) were downloaded directly from NCBI GEO. Only probes that mapped uniquely to human genes using the bioconductor package biomaRt were retained. In cases where multiple probes mapped to the same gene, we selected the probe with the highest mean expression. Differential expression testing of GSE44711 was performed using linear models (lmFit and eBayes functions) and a rotation gene set test (mroast function) in the limma R package [34].

### Gene ontology

Gene lists for each module were tested for enrichment of gene ontology (GO) terms using Fisher exact tests to compute *p*-values for statistical over-representation of GO terms using the GOstats bioconductor package [51] with all the detectable genes (15,861) in our placental gene expression dataset used as the background set.

### Transcription factor motif enrichment

The genes within each co-expression gene module were analysed for enrichment of transcription factor (TF)-binding sites (TFBS) against a background gene set of all detectable genes in the placenta dataset (15,861) using the oPOSSUM program and the JASPAR vertebrate core profiles [52, 53]. For each gene, we searched for TFBS motifs in the conserved regions of the 10kb upstream/downstream sequences using a conservation cut-off of 0.4, a matrix score threshold of 85% and a minimum specificity of 8-bits. The highly enriched TFBSs were identified by ranking TFs using results from Fisher tests and Z-score rankings.

## Additional files

Additional file 1 Supplementary Information. Includes supplementary figures and tables. (PDF 1780 kb)

Additional file 2 Co-expression module gene lists. Comma-seperated values file. This file contains the lists of all the genes in each co-expresion module. (XLSX 85 kb)

Additional file 3 Co-expression module gene ontology terms. Comma-seperated values file. The results of gene ontology analyses for each co-expression module. (XLSX 50 kb)

Additional file 4 Predicted transcription factos for each co-expression module. Comma-seperated values file Top 10 predicted transcription factors for each co-expression module. (XLSX 62 kb)
