# Supplementary material for: Placental transcriptome co-expression analysis reveals conserved regulatory programs across gestation
Source: BMC Genomics. 2017 Jan 3;18:10. doi: 10.1186/s12864-016-3384-9 (PMC5209944; doi:10.1186/s12864-016-3384-9)
Supplement: Additional file 1 — Supplementary Information. Includes supplementary figures and tables. (PDF 1780 kb) [file 12864_2016_3384_MOESM1_ESM.pdf]

Placental transcriptome co-expression analysis reveals conserved regulatory programs across gestation

Sam Buckberry[1], Tina Bianco-Miotto[1,2], Stephen J Bent[1], Vicki Clifton[1], Cheryl Shoubridge[1], Kartik Shankar[3], Gustaaf A Dekker[1], and Claire T Roberts[1,*]

[1]The Robinson Research Institute, The University of Adelaide, School of Paediatrics and Reproductive Health, Adelaide, 5005, Australia
[2]The University of Adelaide, School of agriculture, food and wine, Adelaide, 5005, Australia
[3]University of Arkansas for Medical Sciences, The Department of Pediatrics, Little Rock, 72202, USA
[*]claire.roberts@adelaide.edu.au

Supplementary Information

Table 1. Fetal and maternal clinical variables for placental samples. *Mothers BMI was recoreded at first appointment, at approximately 10 weeks gestation.

Sample ID	Mother's BMI	Fetal sex	Fetal birth-weight (g)	Head circumfer-ence (cm)	Length (cm)	Delivery method	Gestational age (weeks)
1	24.4	Female	3140	33.3	47.9	Operative vaginal	40.4
2	21.9	Female	3050	33.5	47.8	Unassisted vaginal	40.9
3	24.8	Male	3565	34.5	50.4	Unassisted vaginal	39.3
4	20.9	Male	3150	34.8	49.6	Unassisted vaginal	41.1
5	25.2	Female	4010	36	53.0	Unassisted vaginal	41.6
6	26.9	Male	3990	35.8	50.8	Unassisted vaginal	41.4
7	21.6	Male	3464	34.5	49.5	Unassisted vaginal	41.3
8	19.0	Female	3705	35	51.0	Operative vaginal	41.1
9	18.9	Female	3250	33.5	50.5	Cesarean Section in Labor	41.4
10	18.0	Female	3860	35.7	50.2	Cesarean Section in Labor	41.4
11	26.3	Male	3875	36	50.2	Cesarean Section in Labor	40.7
12	25.7	Male	3600	36	49.8	Unassisted vaginal	40.6
13	24.5	Male	4200	36.5	52.9	Operative vaginal	39.9
14	24.6	Female	3550	35.3	49.2	Unassisted vaginal	40.6
15	25.4	Female	3120	33	48.0	Unassisted vaginal	39.9
16	24.0	Male	3680	34.5	48.5	Unassisted vaginal	40.6
Mean	23.3	–	3575.6	34.9	50.0	–	40.8
SD	2.8	–	358.1	1.1	1.6	–	0.6

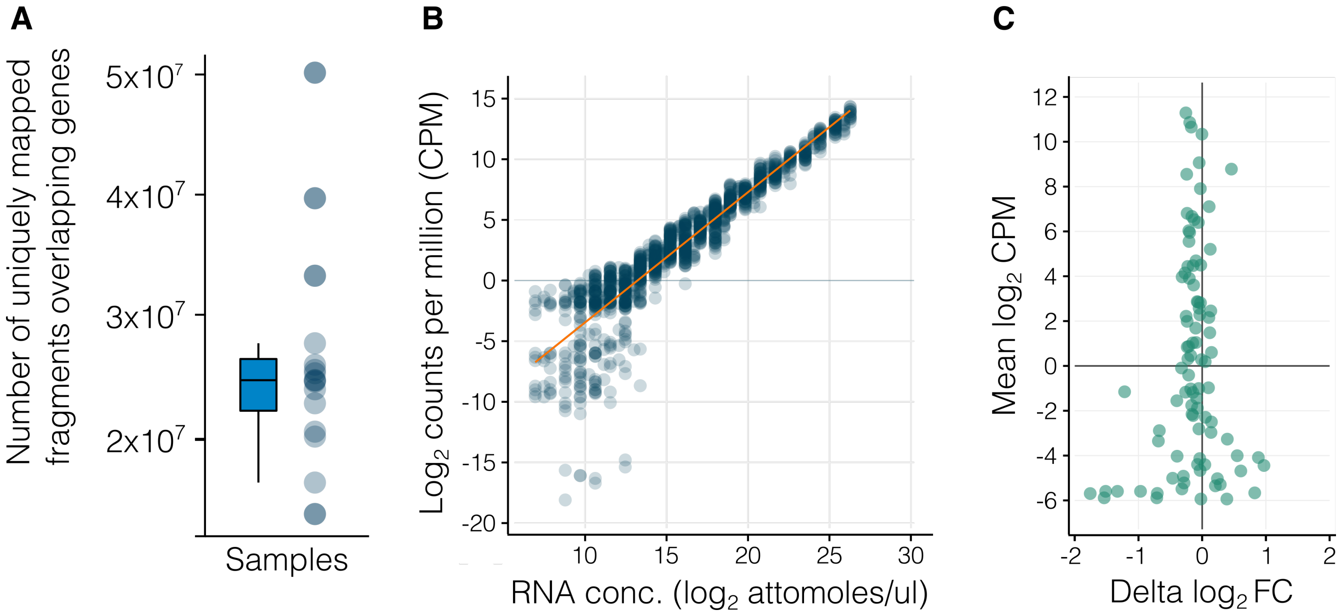

Figure 1. RNA-Seq metrics and quality control. (A) Number of mapped reads per sample. (B) Absolute concentration of ERCC spike-in RNA transcripts is highly correlated with normalised expression above 1 count per million (CPM). (C) Delta fold change (absolute fold difference – the detected fold difference) for ERCC spike-in RNA transcripts (x-axis) versus normalised expression level.

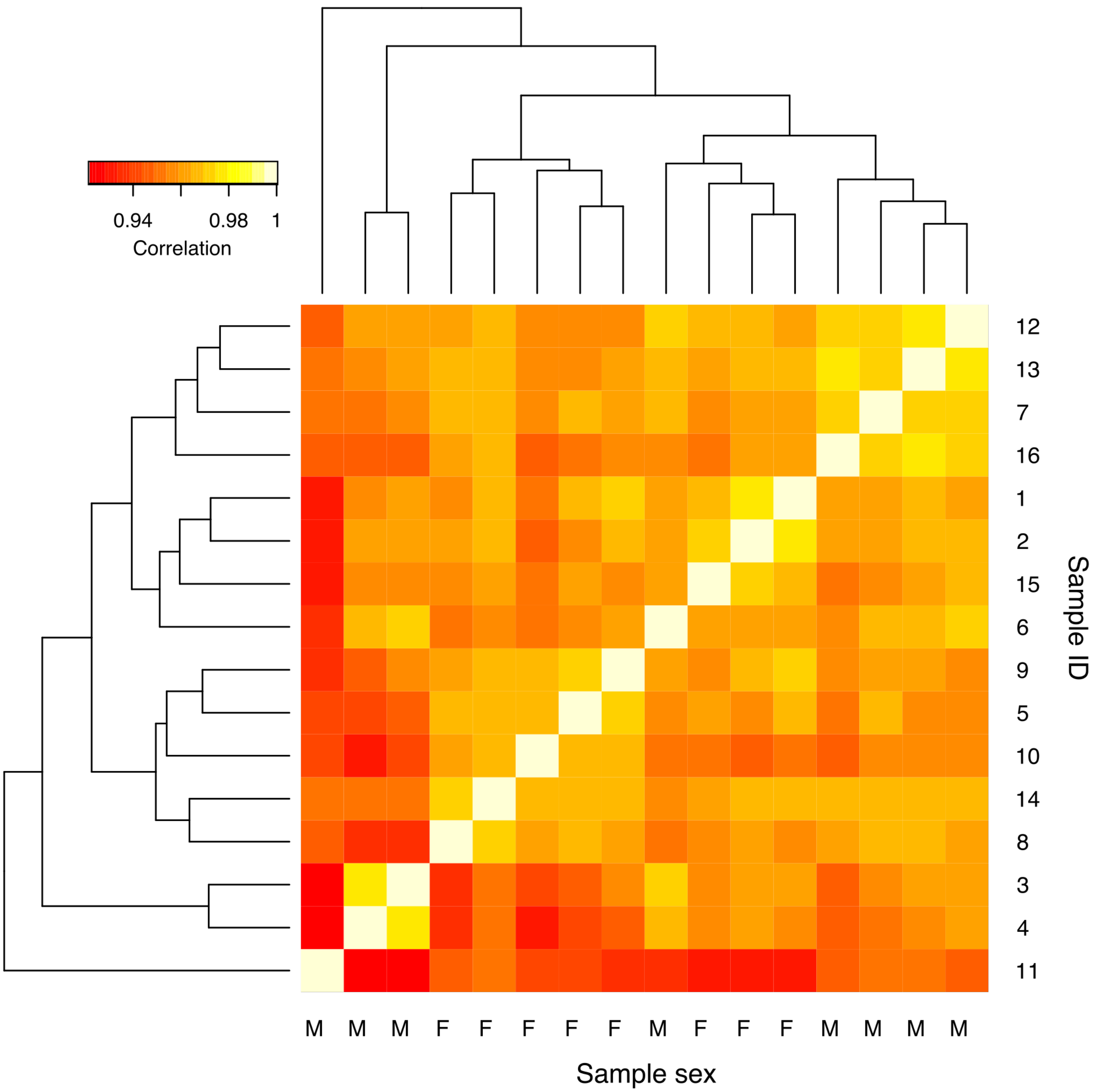

Figure 2. Heatmap showing pairwise correlations of normalized gene expression values. Clustering of samples shows male and female largely group together (x-axis).

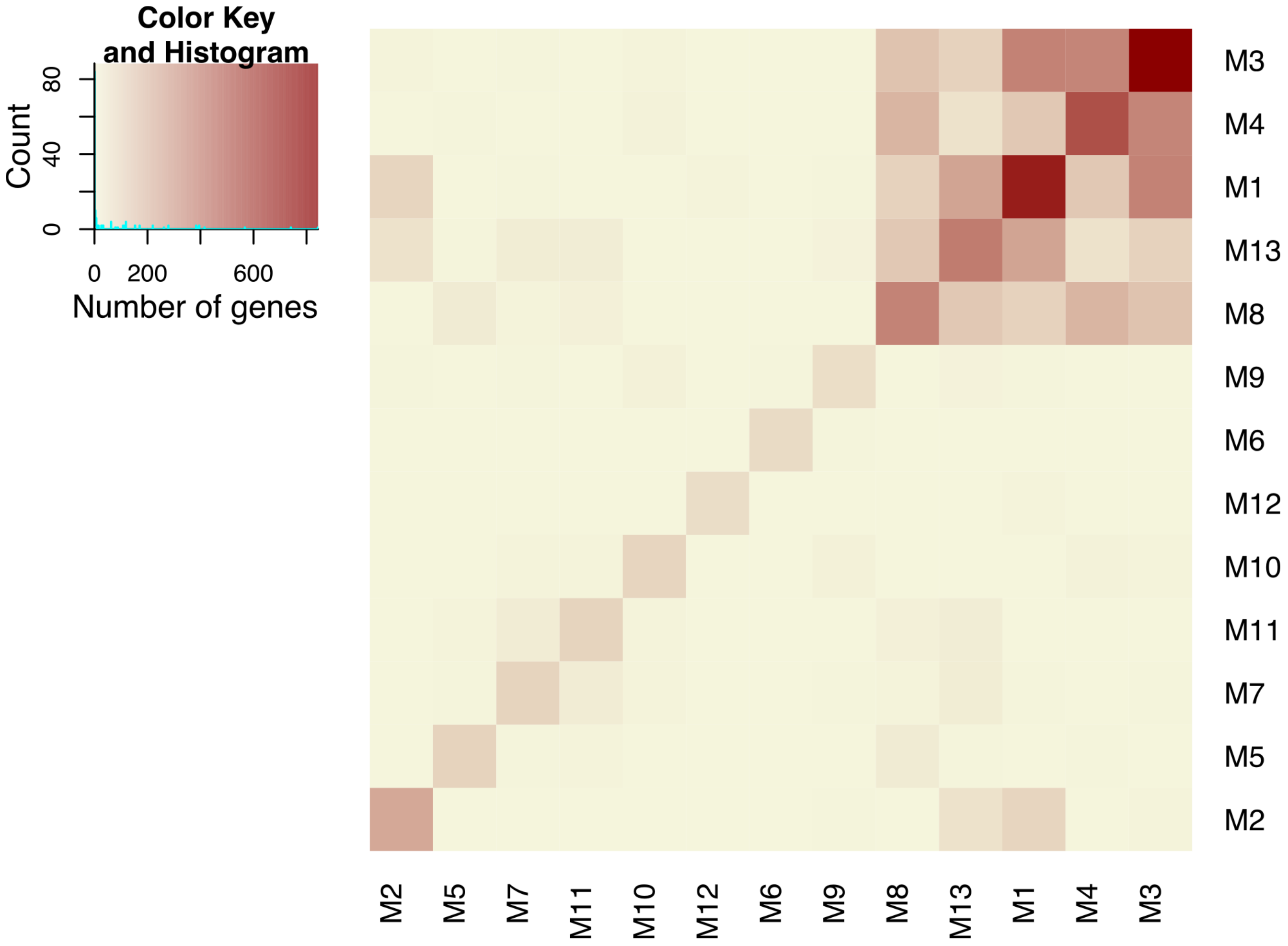

Figure 3. Heat map showing the gene overlap between co-expression modules. Colour intensity represents the number of overlapping genes between two modules.

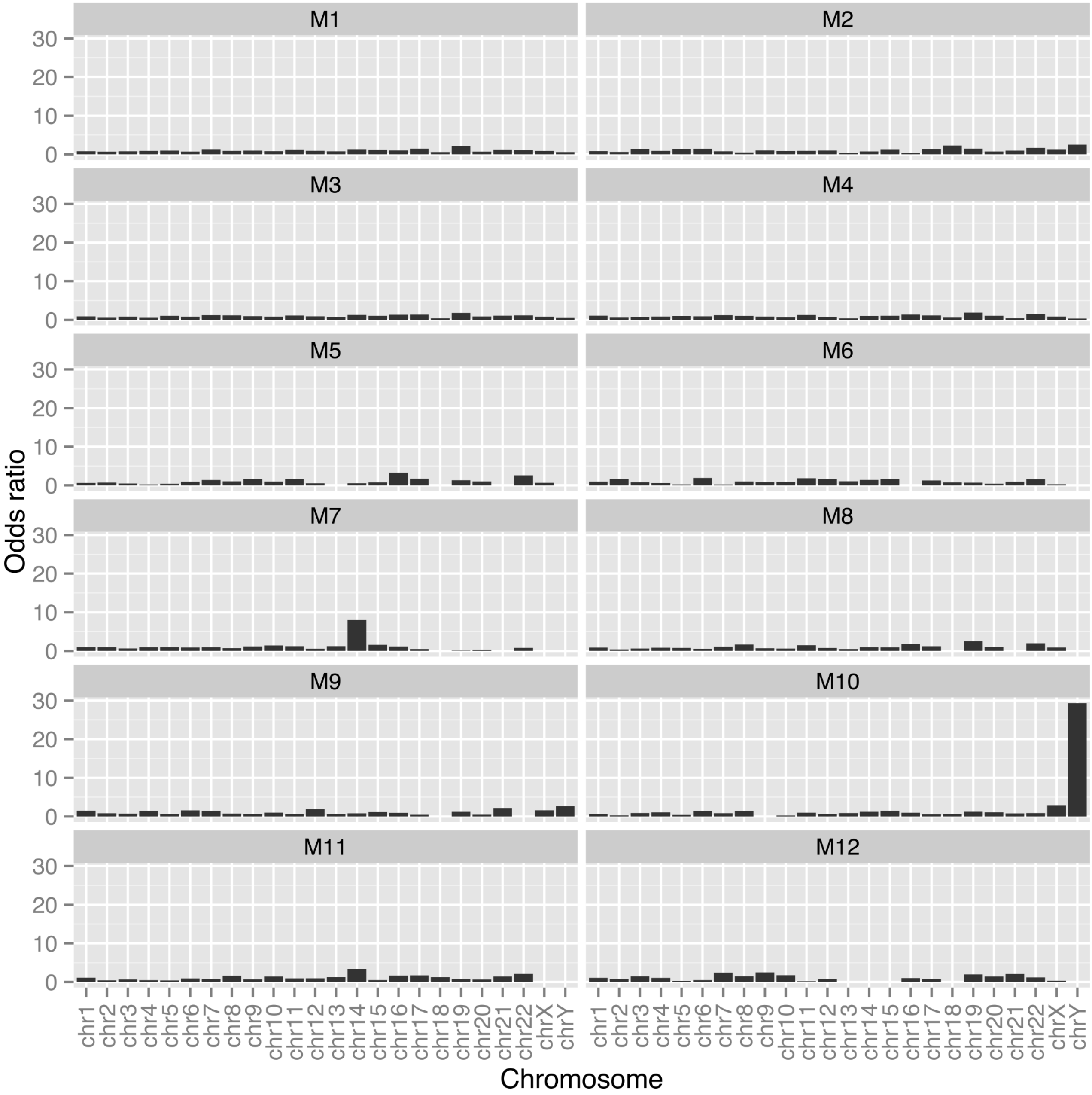

Figure 4. Odds ratios for chromosomal enrichment tests for co-expression modules in the human placenta. These results indicate that the M10 features the most striking enrichment for Y-chromosome genes (Bonferroni $p = 2.9 \times 10^{-12}$, $OR = 29.4$). Each co -expresion module (M1-M12) was tested for over-representation of genes from all autosomes and sex chromosomes with Fisher exact tests.

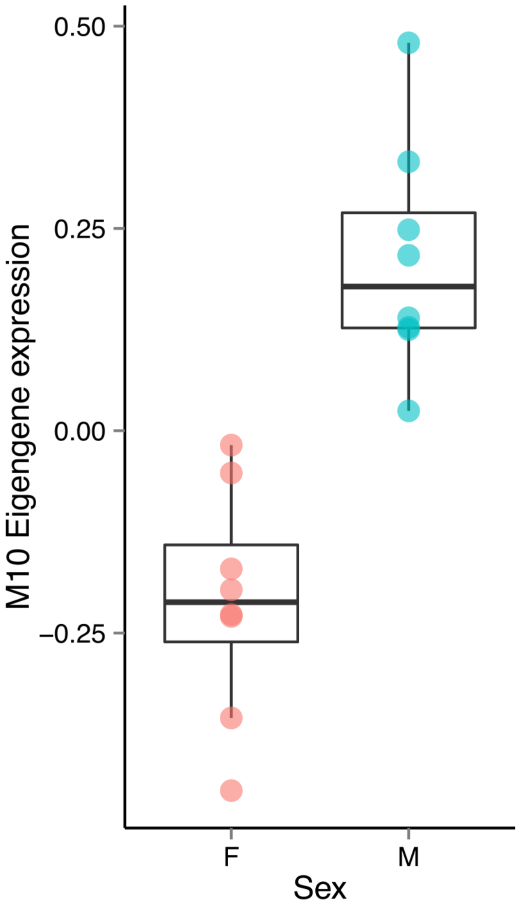

Figure 5. M10 eigengene expression is significantly different between males and females (t-test, $p = 3.5 \times 10^{-5}$, $CI = 0.27 - 0.57$). Plot shows the eigengene values for male and female samples (points) and distribution (boxplots) for the M10 co-expression module in the human placenta.

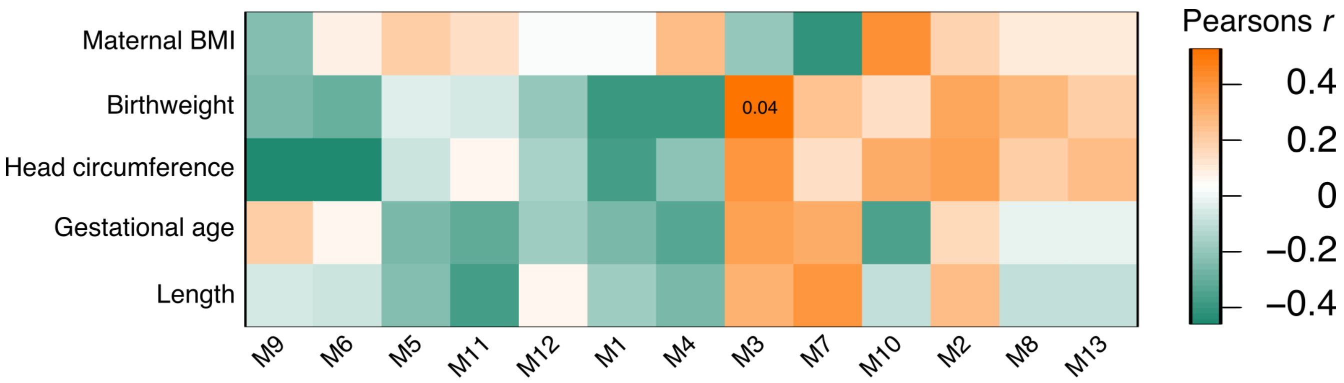

Figure 6. Correlations of module eigengene expression with clinical variables. Heatmap shows Pearson's r value with both variables sorted by hieracrhical clustering. The M3 eigengene was significantly correlated with fetal birthweight (Student asymptotic p=0.038).

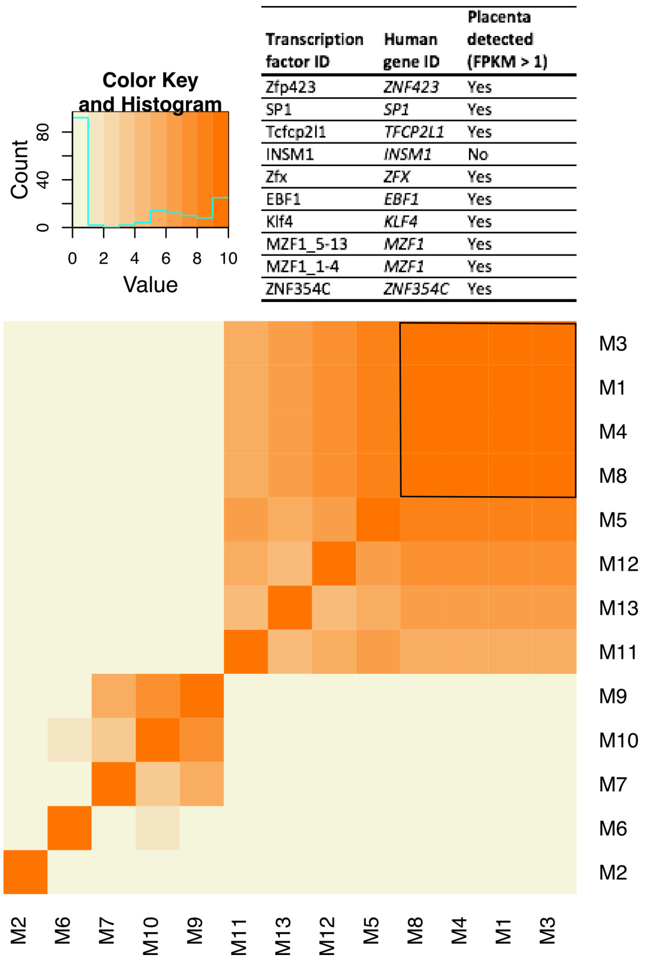

Transcription factor ID	Human gene ID	Placenta detected (FPKM > 1)
Zfp423	*ZNF423*	Yes
SP1	*SP1*	Yes
Tcfcp2l1	*TFCP2L1*	Yes
INSM1	*INSM1*	No
Zfx	*ZFX*	Yes
EBF1	*EBF1*	Yes
Klf4	*KLF4*	Yes
MZF1_5-13	*MZF1*	Yes
MZF1_1-4	*MZF1*	Yes
ZNF354C	*ZNF354C*	Yes

Figure 7. Heat map showing the number of overlapping top ten transcription factors predicted to regulate each co-expression module. The same top ten transcription factors are predicted in M1, M3, M4 and M8 and are shown in the table above heat map.

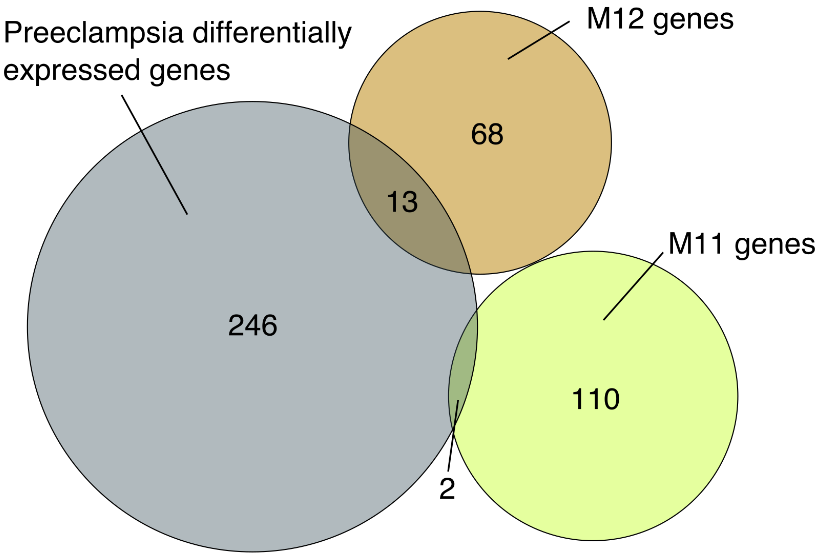

Figure 8. Euler diagram showing the proportion of differentially expressed genes between controls and preeclampsia (data from GSE44711) that are featured in M11 and M12 co-expression modules.

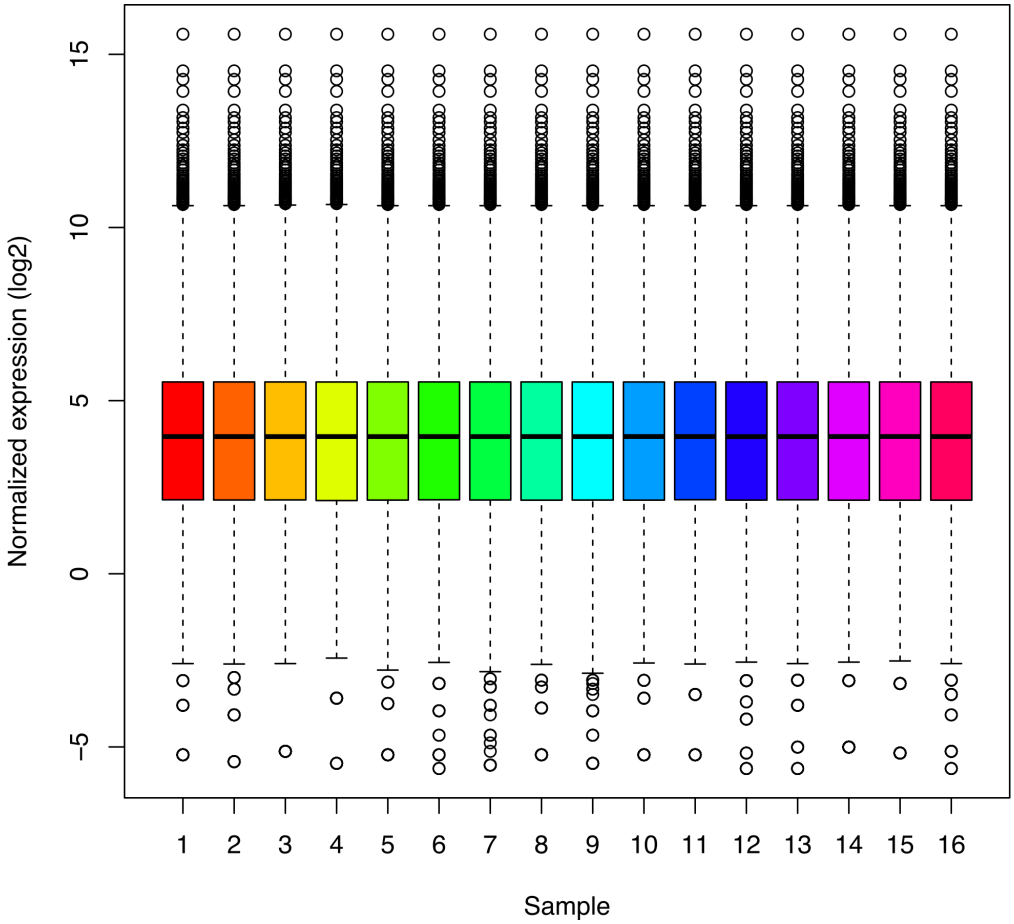

Figure 9. Boxplots showing the distribution of normalized gene expression values across all placental samples. This plot indicates that there are no systematic differences between the samples.

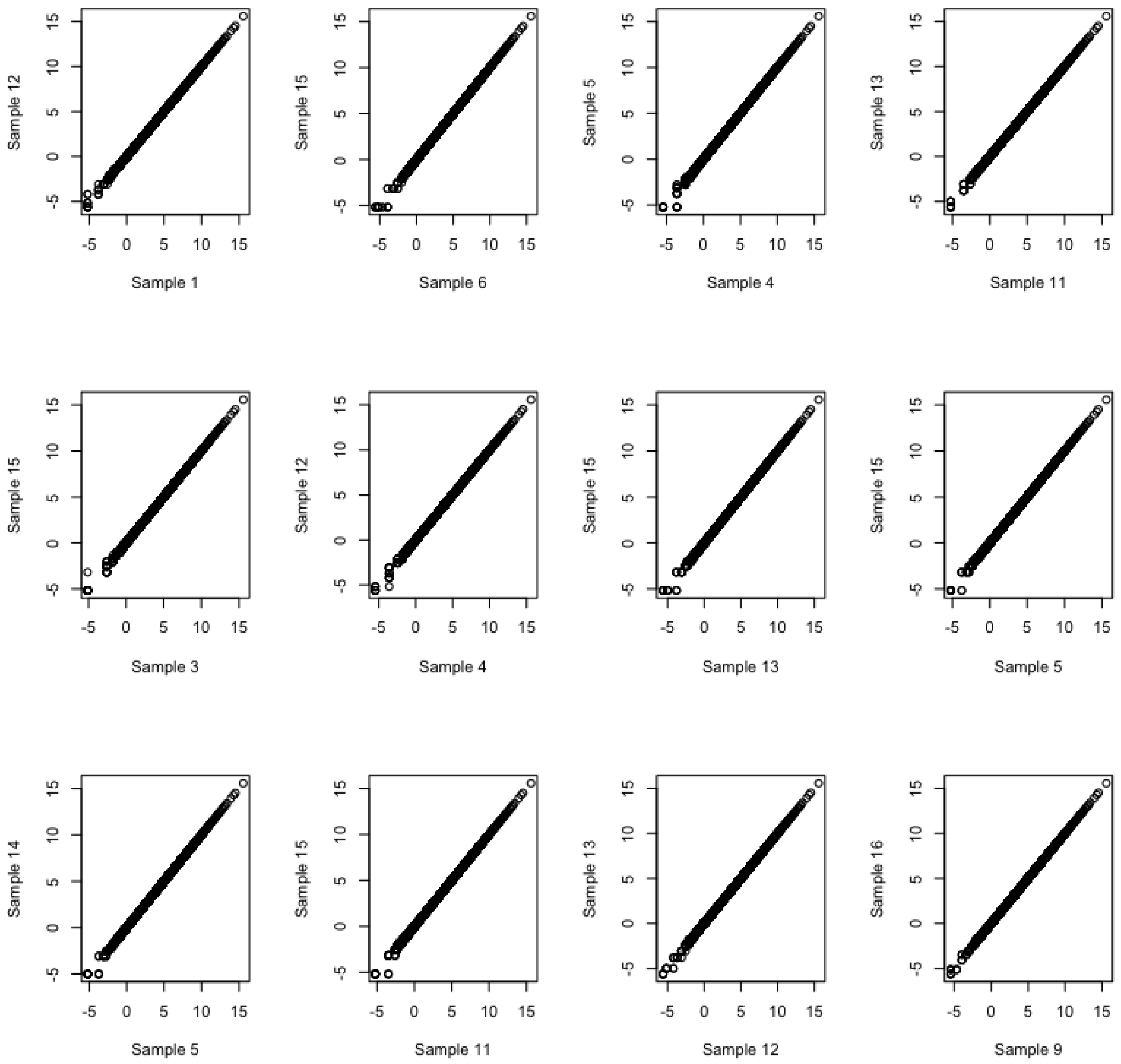

Figure 10. Pairwise sample quantile scatter plots (QQ-plots) of normalized gene expression indicate there are no systematic differences between samples. The presented sample pairs were randomly generated.

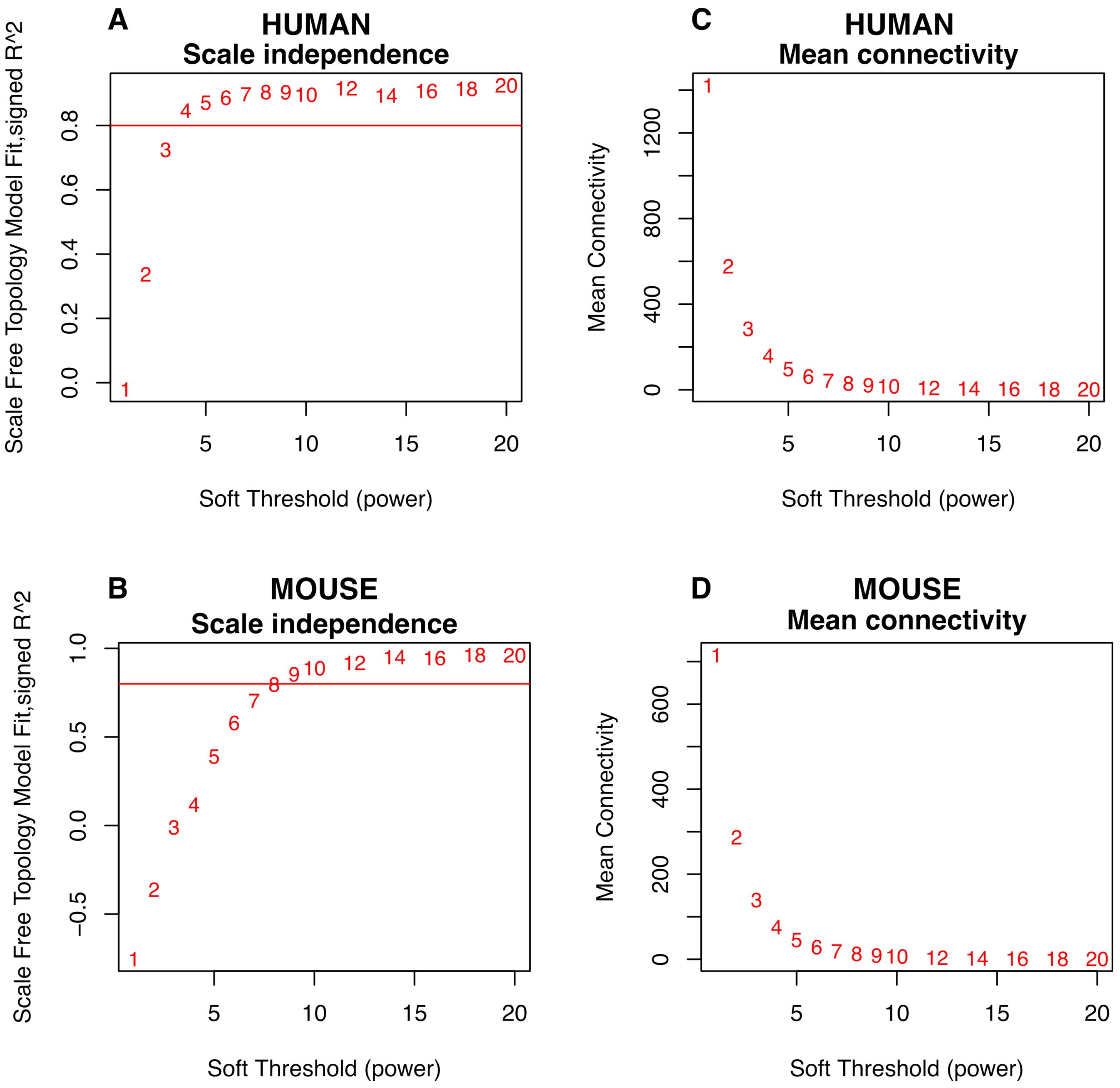

Figure 11. Summary network indices (y-axes) as functions of the soft-thresholding power (x-axes) for human and mouse. Numbers in the plots indicate the corresponding soft-thresholding powers. Plots indicate that approximate scale-free topology is attained bewtween the soft-thresholding power of 6-8 for both datasets.
